# Supplementary material for: Thigh-hip ratio is significantly associated with all-cause mortality among Japanese community-dwelling men
Source: PLoS One. 2023 Oct 5;18(10):e0292287. doi: 10.1371/journal.pone.0292287 (PMC10553839; doi:10.1371/journal.pone.0292287)
Supplement: Attachment — Submitted filename: Response to Reviewers.docx [file pone.0292287.s004.docx]

PONE-D-23-14105

PLOS ONE

**Dear Sir;**

Thank you very much for the valuable suggestions and comments on our manuscript entitled “Thigh-hip ratio is significantly associated with all-cause mortality among Japanese community-dwelling men”. We appreciate your positive suggestions on our manuscript. W

e are submitting here the revised manuscript. We revised it according to all the suggestions made by the reviewers, and the revisions are highlighted in red in the manuscript. Our incorporation of the reviewers’ suggestion is as follows:

If applicable, we recommend that you deposit your laboratory protocols in [protocols.io](http://protocols.io/) to enhance the reproducibility of your results. Protocols.io assigns your protocol its own identifier (DOI) so that it can be cited independently in the future. For instructions see: <https://journals.plos.org/plosone/s/submission-guidelines#loc-laboratory-protocols>. Additionally, PLOS ONE offers an option for publishing peer-reviewed Lab Protocol articles, which describe protocols hosted on [protocols.io](http://protocols.io/). Read more information on sharing protocols at <https://plos.org/protocols?utm_medium=editorial-email&utm_source=authorletters&utm_campaign=protocols>.

Response

Thank you for your advice and we have confirmed them.

Journal Requirements:

Please review your reference list to ensure that it is complete and correct. If you have cited papers that have been retracted, please include the rationale for doing so in the manuscript text, or remove these references and replace them with relevant current references. Any changes to the reference list should be mentioned in the rebuttal letter that accompanies your revised manuscript. If you need to cite a retracted article, indicate the article’s retracted status in the References list and also include a citation and full reference for the retraction notice.

 Response

Thank you for your advice and we have confirmed them.

Additional Editor Comments:

Please clearly and thoroughly address all the comments raised by reviewers

6. Review Comments to the Author

Please use the space provided to explain your answers to the questions above. You may also include additional comments for the author, including concerns about dual publication, research ethics, or publication ethics. (Please upload your review as an attachment if it exceeds 20,000 characters)

Reviewer #1: Additional comments to be addressed
1. Line 92 "The participants' current status, their level of physical activity (e.g., exercise habits)". Revise the sentence as exercise habits is not an example of levels of physical activity (levels of physical activity range from sedentary to vigorous)

Response

Thank you for your advice and we corrected it. (Line 92-93)

The participants' current status, exercise habits, details about their medical history, and medications were collected through a structured questionnaire interview.

2. It is important for the authors to note that exercise habits are not mutually exclusive. In other words, you cannot classify exercise habits as binary "Yes or NO". Levels of physical activity range from sedentary to vigorous. Please revise the classification of levels of physical activity in entire manuscript.

Response

Thank you for your advice and in this study, we classified those who have been exercising continuously for a certain period of time as having an exercise habit, as shown in the examples. Thus, we only have data on the presence or absence of exercise habits. We mentioned its importance in the limitations of the study. (Line 92-98; Line 385-387)

To qualify as having exercise habits, the participants must have participated in any type of moderate-to-vigorous physical activity, such as brisk walking, golfing, gardening, jogging, or playing tennis, for at least 30 minutes on at least 2 days per week (≥ 600 MET-minutes/week) for a minimum of 1 year.

Fourth, there was a lack of direct and detail data concerning exercise habits, muscle mass, and body composition which are important for maintaining physique and health.

3. Line 122-128 the paragraph is not clear.

Response

Thank you for your advice and we corrected it. (Line 125-131)

Participants were asked to fast overnight before measurements and their triglycerides (TG), high-density lipoprotein cholesterol (HDL-C), low-density lipoprotein cholesterol (LDL-C), serum uric acid (SUA), and creatinine (Cr) (enzymatic assay, Kyowa Medex, Tokyo, Japan) were measured using an automated analyzer (Hitachi, Tokyo, Japan). Hemoglobin A1c (HbA1c) was analyzed by the latex agglutination inhibition method (Kyowa Medex, Tokyo, Japan) using an automated analyzer (DM-JACK® Ex, Tokyo, Japan).

4. Line 141 "Participants were classified as having hypertriglyceridemia if their TG level was < 150 mg/dL" This is not correct.

Response

Thank you for your advice and we corrected it. (Line 144)

.......and hypertriglyceridemia if their TG level was ≥ 150 mg/dL.

Reviewer #2: Reviewer Comments for the revised manuscript
1. ABSTRACT
Well summarized

Response：Thank you for your comment.

2. INTRODUCTION
Narrowed to the aim of the study

Response：Thank you for your comment.

3. MATERIAL AND METHODS
Study design and participants
3.1. Line 92-97 is clear but does not quantify the level of physical activity to meet the minimum requirement.

Response

Thank you for your advice but in this study, we classified those who have been exercising continuously for a certain period of time as having an exercise habit, as shown in the examples. Thus, we only have data on the presence or absence of exercise habits. We mentioned its importance in the limitations of the study. (Line 385-387)

Fourth, there was a lack of direct and detail data concerning exercise habits, muscle mass, and body composition which are important for maintaining physique and health.

3.2. The brand, model, and manufacturer of the instrument used are not stated (the weighing scale and the stadiometer) in lines 99 and 101. Also, the instrument for measuring waist, hip, and thigh circumference is not stated, in lines 103-106

Response

Thank you for your advice and we inserted them. (Line 99-105)

Body weight was measured to the nearest 0.1 kg with an electronic standard weight scale machine (HBF-214, Omron, Tokyo, Japan), with participants wearing underwear or light clothing and body height was measured without shoes to the nearest 1 cm using a stadiometer (DSN-90, Muratec-KDS, Kyoto, Japan). Body mass index (BMI) was determined as weight (kg) divided by squared height (m^2^). Waist, hip, and thigh circumferences (cm) were measured with an anthropometric tape (25-204, Clover, Osaka, Japan).

4. Evaluation of risk factors

4.1. Line 107-108 well revised and is clear

Response：Thank you for your comment.

4.2. Lines 118-122 are well revised, but the positioning of participants is not stated, can the author state the position

Response

Thank you for your advice and we corrected it. (Line 121-125)

Systolic and diastolic blood pressure (SBP and DBP, respectively) were measured using an automated sphygmomanometer (BP-103i; Colin, Aichi, Japan) with an appropriately sized cuff placed on their right upper arm while the subjects were seated after at least 5 minutes of immobility, and the average of two consecutive measurements was used for calculation.

4.3. Line 145-146 is clear cardiovascular disease

Response：Thank you for your comment.

5. STATISTICAL ANALYSIS
5.1. Lines 383-387 state the limitation of the study of categorizing BMI of 25 or greater

Response：Thank you for your comment.

5.2. Lines 92-97: explanation is clear but does not quantify the level of physical activity to meet the minimum requirement, the recommended is 600MET/MIN/WEEK for healthy living

Response

Thank you for your advice but in this study, we classified those who have been exercising continuously for a certain period of time as having an exercise habit, as shown in the examples. Thus, we only have data on the presence or absence of exercise habits. We mentioned its importance in the limitations of the study. (Line 94-98; Line 385-387)

To qualify as having exercise habits, the participants must have participated in any type of moderate-to-vigorous physical activity, such as brisk walking, golfing, gardening, jogging, or playing tennis, for at least 30 minutes on at least 2 days per week (≥ 600 MET-minutes/week) for a minimum of 1 year.

Fourth, there was a lack of direct and detail data concerning exercise habits, muscle mass, and body composition which are important for maintaining physique and health.

5. RESULTS
5.1. Lines 270-272, 244-246, 240-273,270: are clear well revised the comments put forth on the previous manuscript

Response：Thank you for your comment.

6. DISCUSSION
Lines 312-316 are clear and understood
Lines 383-387 are clear are limitation factors for BMI categorization
Line 276-283,286-297: noted for elaboration on the confounders

Response：Thank you for your comment.

7. CONCLUSION
Line 393-399: noted for the modification done.

Response：Thank you for your comment.

In summary, we appreciate your careful reading of our manuscript and instructive comments. We believe that our revised manuscript has been improved by the incorporation your suggestions. We hope that the changes will meet with your approval.
